# Switching specific biomolecular interactions on surfaces under complex biological conditions†
†Electronic supplementary information (ESI) available. See DOI: 10.1039/c4an01225a



**DOI:** 10.1039/c4an01225a

**Published:** 2014-09-02

**Authors:** Minhaj Lashkor, Frankie J. Rawson, Jon A. Preece, Paula M. Mendes

**Affiliations:** a School of Chemical Engineering , University of Birmingham , Edgbaston , Birmingham , B15 2TT , UK . Email: p.m.mendes@bham.ac.uk; b Laboratory of Biophysics and Surface Analysis , School of Pharmacy , University of Nottingham , University Park , Nottingham , NG 72RD , UK; c School of Chemistry , University of Birmingham , Edgbaston , Birmingham , B15 2TT , UK

## Abstract

Herein, electrically switchable mixed self-assembled monolayers based on oligopeptides have been developed and investigated for their suitability in achieving control over biomolecular interactions in the presence of complex biological conditions. Our model system, a biotinylated oligopeptide tethered to gold within a background of tri(ethylene glycol) undecanethiol, is ubiquitous in both switching specific protein interactions in highly fouling media while still offering the non-specific protein-resistance to the surface. Furthermore, the work demonstrated that the performance of the switching on the electro-switchable oligopeptide is sensitive to the characteristics of the media, and in particular, its protein concentration and buffer composition, and thus such aspects should be considered and addressed to assure maximum switching performance. This study lays the foundation for developing more realistic dynamic extracellular matrix models and is certainly applicable in a wide variety of biological and medical applications.

## Abstract 2

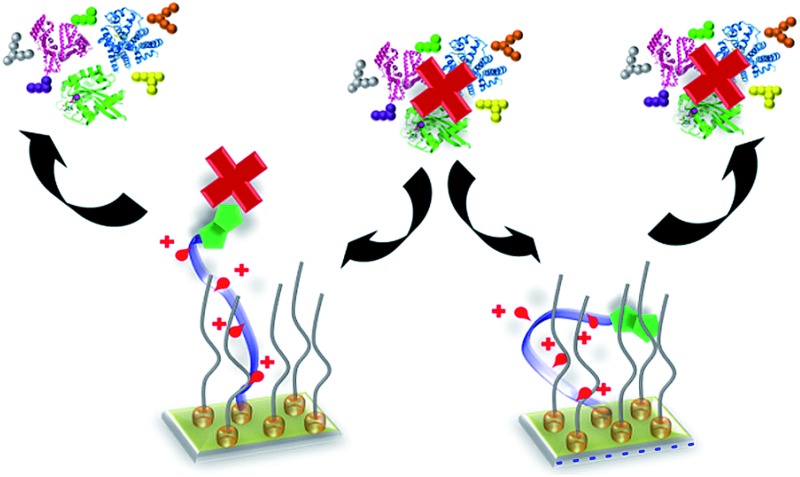
Electrically switchable surfaces based on oligopeptides are ubiquitous in both switching specific protein interactions in highly fouling media while still offering the non-specific protein-resistance to the surface.

## Introduction

Stimuli-responsive surfaces that can regulate bimolecular interactions on-demand are enabling novel functionalities and new device designs for a variety of biological and medical applications.^1,2^ These responsive surfaces offer intriguing possibilities in the development of highly sensitive biosensors,^3–6^ novel drug delivery systems^7–9^ and highly functional microfluidic, bioanalysis, and bioseparation systems.^10–13^ Additionally, dynamic, synthetic surfaces that can control the presentation of regulatory signals to a cell are expected to have a significant impact in the field of tissue engineering and regenerative medicine, and to provide unprecedented opportunities in fundamental studies of cell biology.^14,15^

A key challenge yet to be fulfilled by switchable surfaces is to regulate, and understand, specific biomolecular interactions that are driven by external stimuli in complex biological conditions. This will ensure that biological information and control generated through such tools mimic the natural biological environment. Biological systems are typically a complex mixture of inorganic salts, inorganic complexes, amino acids, peptides, and proteins.^16^ The majority of studies on stimuli-responsive surfaces reported to date either rely on controlling non-specific interactions (*i.e.*, hydrophobic/hydrophilic and electrostatic) of the biomolecules with the active surface,^17–21^ or have focused on demonstrating modulation of specific biomolecular interactions under simple biological conditions, typically water or buffer solutions.^22–24^ For instance, temperature control over specific interactions in water have been achieved by using a two-component mixed self-assembled monolayer (SAM) on gold comprising oligo(ethylene glycol) (OEG) thiol molecules and shorter disulfides carrying biotin end-groups.^22^ The OEG thiols were able to switch in response to a change in temperature below and above their lower critical solution temperature (LCST = 37 °C). At 23 °C the structure of the OEG molecules was fully extended hindering the shorter biotin disulfide components. On the contrary, at 45 °C the OEG backbone collapsed, thus allowing the specific interaction between the biotin molecule on the surface and the protein streptavidin in solution.

In our recent work,^25^ we have demonstrated that oligopeptides, which bear at one extremity a functional group able to anchor to a substrate and a bioactive molecular moiety at the other extremity, can act as functional components on switchable surfaces for controlling specific biomolecular interactions. The system is based upon the conformational switching of positively charged oligolysine peptides which are tethered to a gold surface, such that bioactive molecular moieties incorporated on the oligolysines can be reversibly exposed (bio-active state) or concealed (bio-inactive state) on demand, as a function of surface potential.^26,27^

Previously,^25^ we have tested the operation of switchable oligopeptides on mixed SAMs on gold surfaces to control biomolecular interactions under only very limited biological conditions (*i.e.* phosphate buffer saline – PBS) using an electrical potential as the actuator. These SAMs have been shown to regulate the binding between the biotin ligand on the surface and neutravidin from solution. Switchable SAMs used to control biomolecular interactions *via* an electrical stimulus are particularly appealing because of their fast response times, ease of creating multiple individually addressable switchable regions on the same surface, as well as low-drive voltage and electric fields, which are compatible with biological systems.^28^ These inherent properties, along with the diversity of bioactive molecular entities which can be chemically attached to the oligopeptide, make these oligopeptide SAMs excellent candidates to realize high performance electrically switchable surfaces for complex biological conditions.

**Fig. 1:**
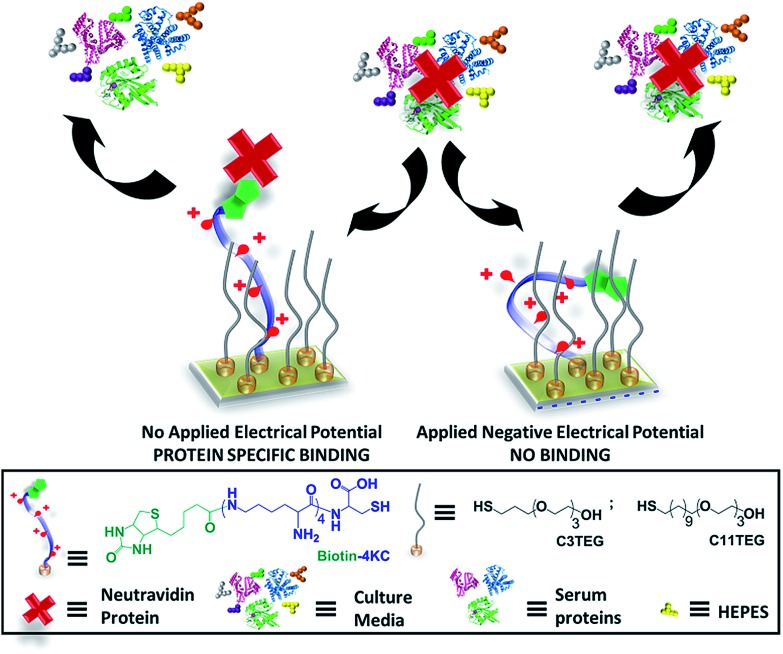
Schematic of the dynamic oligopeptide SAM proposed for controlling specific biomolecular interactions under complex biological conditions. Below: chemical structures of the oligopeptide (**biotin-4KC**) and oligo(ethylene glycol) thiols (**C3TEG** and **C11TEG**) used for SAM preparation.

Although development of surfaces with switching functions under complex biological conditions is highly desirable from the standpoint of biomedical applications, studies to such effect are scarce^29–32^ and more investigations are clearly needed to understand and develop molecular-based platforms to address solutions within the biomedical field. To address the challenge of developing and understanding new switchable surfaces, in this paper we have conducted a detailed study, using electrochemical surface plasmon resonance (SPR) spectroscopy, on the influence of the characteristics of complex biological medium (both its chemical and protein composition and its inherent physicochemical properties) on the switchable properties of a oligopeptide SAM model system (Fig. 1).

The switchable oligopeptide SAM model system was composed of two molecular components: (i) a positively charged 4-mer of lysine (K) that is functionalized at one end with biotin, which recognises the neutravidin protein, and at the other end with a cysteine (C) for binding to gold substrates *via* the thiol group (**biotin-4KC**), and (ii) an ethylene glycol-terminated thiol (**C11TEG**). The interaction of the neutravidin protein to a surface appended biotin ligand was chosen for these studies presented here because it can be easily monitored and quantified by SPR, allowing for facile evaluation of the switching performance under various biological conditions. Aiming at cellular applications, which require complex culture media, we have then evaluated the switching properties in three different commonly used media for cell and tissue culture, namely Dulbecco's Modified Eagle Medium (DMEM), DMEM containing 10% fetal bovine serum (DMEM–FBS), with protein levels of approximately 3.2 mg ml^–1^, and DMEM–FBS with 24 mM (4-(2-hydroxyethyl)-1-piperazineethanesulfonic acid) HEPES buffer (DMEM–FBS–HEPES). Note that DMEM contains a mixture of inorganic salts, amino acids, glucose and vitamins.

## Experimental

### Chemicals and materials

Commercially available chemicals and solvents were purchased from Aldrich Chemicals and Fisher Chemicals and were used as received. The oligopeptide **biotin-4KC** was synthesised by Peptide Protein Research Ltd. (Wickham, UK) to >95% purity and verified by HPLC and mass spectrometry. The (3-mercaptopropyl)tri(ethylene glycol) (**C3TEG**) was prepared as previously described.^25^ The (11-mercaptoundecyl)tri(ethylene glycol) (**C11TEG**), fetal bovine serum (FBS) and 4-(2-hydroxyethyl)-1-piperazineethanesulfonic acid (HEPES) buffer (1 M) were purchased from Sigma Aldrich and used as received. Neutravidin and DMEM were obtained from Invitrogen Life Technologies. Phosphate buffered saline (PBS) solution was prepared from a 10× concentrate PBS solution (1.37 M sodium chloride, 0.027 M potassium chloride, and 0.119 M phosphate buffer) from Fisher BioReagents. DMEM–FBS contains DMEM with 10% (v/v) FBS. DMEM–FBS–HEPES contains 10% FBS and 24 mM HEPES. Polycrystalline gold substrates were purchased from George Albert PVD, Germany and consisted either of a 50 nm gold layer deposited onto glass covered with a thin layer of chromium as the adhesion layer (used for contact angle and XPS analysis) or 100 nm gold layer on 100-4 inch-silicon wafer, precoated with titanium as the adhesion layer (for ellipsometry analysis). SPR gold chips were purchased from Reichert Technologies, US.

### Preparation of mixed self-assembled monolayers (SAMs)

The Au substrates were cleaned by immersion in piranha solution (3:1, H_2_SO_4_: 30% H_2_O_2_) at room temperature for 10 min, rinsing with Ultra High Pure (UHP) H_2_O and then HPLC grade EtOH thoroughly for 1 min. (*Caution: Piranha solution reacts violently with all organic compounds and should be handled with care*). For the preparation of the **biotin-4KC**:**C3TEG** or **biotin-4KC**:**C11TEG**, mixed SAMs, solutions of the oligopeptide (0.1 mM) and either **C3TEG** or **C11TEG** (0.1 mM) were prepared in HPLC EtOH containing 3% (v/v) N(CH_2_CH_3_)_3_, and mixed at the volume ratio of 1 : 40. Subsequently, the clean Au substrates were immersed in the mixed solutions for 24 h to form the mixed SAMs on the Au surfaces. The substrates were rinsed with HPLC EtOH, an ethanolic solution containing 10% (v/v) CH_3_COOH, and UHP H_2_O. Note that the mixed SAMs were deposited in the presence of N(CH_2_CH_3_)_3_ to prevent the formation of hydrogen bonds between the NH_2_ functional groups of the bound thiolate peptide on the Au surface and that of free thiol peptide in the bulk solution.^33^ The pure **C3TEG** or **C11TEG** SAMs were prepared by immersing the clean gold substrates in ethanolic 0.1 mM solution of the respective ethylene glycol thiols for 24 h, followed by rinsing with HPLC EtOH.

#### X-ray photoelectron spectroscopy (XPS)

XPS spectra were obtained on the VG Escalab 250 instrument based at University of Leeds EPSRC Nanoscience and Nanotechnology Facility, UK. XPS experiments were carried out using a monochromatic Al Kα X-ray source (1486.7 eV) and a take-off angle of 15°. High-resolution scans of N 1s and S 2p were recorded using a pass energy of 150 eV at a step size of 0.05 eV. Fitting of XPS peaks was performed using the Avantage V 2.2 processing software. Sensitivity factors used in this study were: S 2p, 2.08; N 1s, 1.73; C 1s, 1.00; O 1s, 2.8; Au, 4f_7/2_, 9.58 and Au, 4f_5/2_, 7.54. The averages and standard errors reported were determined from four different XPS measurements.

#### Ellipsometry

The thickness of the deposited monolayers was determined by spectroscopic ellipsometry. A Jobin-Yvon UVISEL ellipsometer with a xenon light source was used for the measurements. The angle of incidence was fixed at 70°. A wavelength range of 280–820 nm was used. The DeltaPsi software was employed to determine the thickness values and the calculations were based on a three-phase ambient/SAM/Au model, in which the SAM was assumed to be isotropic and assigned a refractive index of 1.50. The thickness reported is the average of six measurements, with the errors reported as standard deviation.

#### Contact angle

Contact angles were determined using a home-built contact angle apparatus, equipped with a charged coupled device (CCD) KP-M1E/K camera (Hitachi) that was attached to a personal recorded as a micro-syringe was used to quasi-statically add water to or remove water from the drop. The drop was shown as a live video image on the PC screen and the acquisition rate was 4 frames per second. FTA Video Analysis software v1.96 (First Ten Angstroms) was used for the analysis of the contact angle of a droplet of UHP H_2_O at the three-phase intersection. The averages and standard errors of contact angles were determined from five different measurements made for each type of SAM.

#### Surface plasmon resonance (SPR)

SPR switching experiments were performed with a Reichert SR7000DC Dual Channel Spectrometer (Buffalo, NY, USA) at 25 °C using a three-electrode electrochemical cell and a Gamry PCI4/G300 potentiostat. The SAMs prepared on Reichert Au sensor chips served as the working electrode, the counter electrode was a Pt wire, and a standard calomel electrode (SCE) was used as the reference electrode. Prior to the binding studies, the sensor chips were equilibrated with degassed PBS, followed by application of –0.4 V or open circuit conditions for 10 min while passing degassed PBS through the electrochemical cell at a flow rate of 50 μl min^–1^. While still applying a potential, neutravidin (500 μl, 54.4 μg ml^–1^) or neutravidin with DMEM, DMEM–FBS or DMEM–FBS–HEPES were injected over the sensor chip surface for 10 s at 1500 μl min^–1^ and then 30 min at 8 μl min^–1^ (the decrease in flow rate from 1500 to 8 μl min^–1^ ensures that sufficient exposure time is provided for binding to occur between the biotin on the surface and neutravidin in solution). In order to remove any unbound material, the sensor chips were washed with degassed PBS for 10 s at a flow rate of 1500 μl min^–1^, followed by 10 min at a flow rate of 50 μl min^–1^ while still applying a potential to the chips. The same procedure was used for OC experiments without applying a potential.

## Results and discussion

**Fig. 2:**
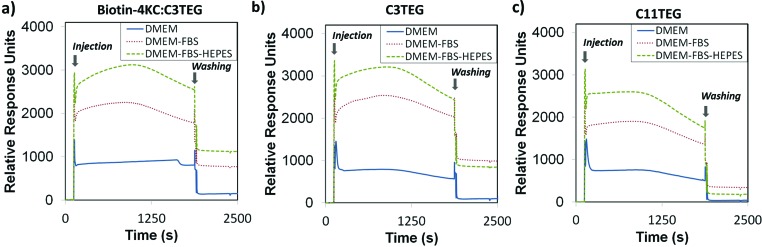
SPR sensorgram traces for the interaction of (a) **biotin-4KC**:**C3TEG**, (b) **C3TEG** and (c) **C11TEG** SAMs with different complex media – DMEM, DMEM–FBS, and DMEM–FBS–HEPES. The two arrows in the graphs indicate the point of injection of media and PBS washing buffer, respectively.

In our previous studies,^25^ the electrically switchable oligopeptide surfaces were based on a two component mixed SAM formed from **biotin-4KC** and a short tri(ethylene glycol)-terminated thiol – (3-mercaptopropyl)tri(ethylene glycol) (**C3TEG**) (Fig. 1). The **C3TEG** was utilized to ensure sufficient spatial freedom for molecular reorientation of the surface bound biotinylated peptide as well as to stop non-specific protein adsorption to the surface. Initial investigations in this paper were performed to elucidate the interactions of the different complex media – DMEM, DMEM–FBS, and DMEM–FBS–HEPES – with the **biotin-4KC**:**C3TEG** mixed SAM. We assessed the non-specific adsorption from three different media on the **biotin-4KC**:**C3TEG** (Fig. 2a) mixed SAMs and the **C3TEG** (Fig. 2b) and **C11TEG** (Fig. 2c) pure SAMs and analysed the adsorption response by SPR. The characterization of these SAMs by contact angle and ellipsometry are outlined in detail in the ESI, Table S1.† In the SPR experiments, the SAMs were exposed to an initial flow of PBS solution, to establish the baseline, followed by an injection (first arrow on left in Fig. 2a–c) of either DMEM, DMEM–FBS or DMEM–FBS–HEPES media into the SPR flow cell for 1800 s (*i.e.* 30 min) at a flow rate of 8 μl min^–1^. The SPR flow cell was then flushed with PBS to remove any physisorbed material, at *t* = 1800 s (second arrow in Fig. 2a–c).

Upon initial injection of the three different media over all three surfaces, there was a rapid response due to differences in the refractive indices of the PBS buffer and three media solutions, followed by a slower increase for **C3TEG** containing SAMs (Fig. 2a and b) as proteins adsorb to the surface, or no further increase for **C11TEG** containing SAMs (Fig. 2c) over the time frame *t* = 10 s to *t* = 1000 s. This result suggests that the **C11TEG** is more resistant to protein adsorption than the shorter **C3TEG**. Between *t* = 1000 s and injection of the PBS buffer there is a decline in the SPR signal for the **C3TEG** containing SAMs (Fig. 2a and b) and the pure **C11TEG** SAM (Fig. 2c), which suggest that initial adsorption of material is reversing, or a reorganization of the adsorbed proteins is occurring. On washing with PBS buffer (*t* = 1800 s, arrow on the right in Fig. 2a–c) into the flow cell, there was an immediate drop in the SPR signal due to the change in the bulk refractive index, for all SAMs. However, the signals remained elevated compared to the original baseline at *t* = 0, but with the smallest elevation for the **C11TEG** containing SAMs, which were about a 33% of the **C3TEG** containing SAMs.

The surface plasmon resonance (SPR) results indicate that the **biotin-4KC**:**C3TEG** mixed SAM is not inert to non-specific serum adsorption, which is expected to be detrimental to the specificity and efficiency of the switching system. Furthermore, the nonspecific response would hinder the evaluation of the switching performance since the adsorption of serum on the SAM surface would lead to a non-specific SPR response, which would be difficult to distinguish from the response resulting from the specific biomolecular interactions.

It is believed that oligo(ethylene glycol) (OEG)-terminated alkanethiolate SAMs resist protein adsorption from solution *via* two possible mechanisms:^34–38^ (i) steric repulsion, resulting from compression of OEG chains as protein approaches the surface and (ii) water barrier due to the formation of strong hydrogen bonds between the oxygen atoms in the ethylene oxide units and the hydrogen atoms in the water molecules. From these two proposed mechanisms, and as demonstrated by previous studies,^34–38^ the molecular conformation and spatial arrangement of the OEG moieties, as well as OEG surface density play an important role in imparting protein resistance. For instance, OEG SAMs have been shown to adsorb proteins when their surface OEG densities were too high or too low, yet non-fouling at appropriate OEG densities.^39^ From these previous reports, it seems reasonable to infer that the presence of a short alkyl chain (*i.e.* C3 (propyl)) between the thiol group and the TEG moiety give disordered low density structures on the gold surface, which allow serum to adsorb to the surface. This is perhaps not surprising, as previous studies^40^ have shown that the structure of short-chain *n*-alkyl thiol assemblies is more disordered than that of the longer chain (above C9) assemblies. Thus, a longer alkyl chain between the thiol group and the TEG moiety was utilized (**C11TEG**, Fig. 1) in this study to achieve a higher density SAM, relative to **C3TEG** to inhibit serum adsorption. The more ordered **C11TEG** SAM was verified by the contact angle hysteresis experiments as discussed in the ESI.†

**Fig. 3:**
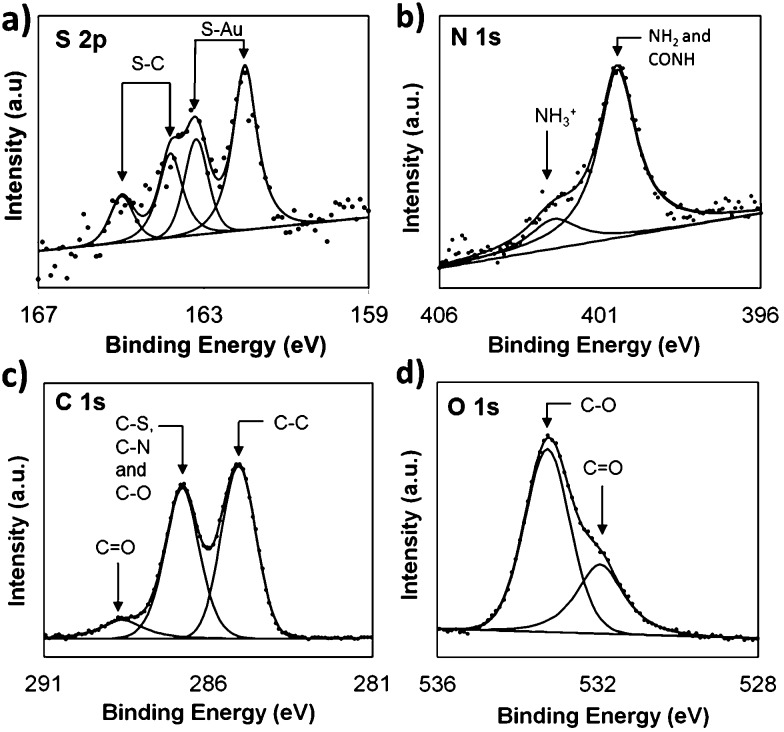
XPS spectra of the (a) S 2p, (b) N 1s, (c) C 1s and (d) O 1s peak regions of **biotin-4KC** : **C11TEG** mixed SAMs at a 1 : 40 solution ratio.

The **biotin-4KC** : **C11TEG** mixed SAMs were formed from a solution ratio of 1 : 40 over 24 hours and characterized by contact angle, ellipsometry (outlined in detail in the ESI†) and X-ray photoelectron spectroscopy (XPS). XPS analysis revealed the presence of the elemental species S, N, C and O on the **biotin-4KC**:**C11TEG SAM** (Fig. 3), confirming thus the formation of the mixed SAM.

The S 2p spectrum (Fig. 3a) consists of two doublet peaks, with one doublet peak at 163.2 eV (S 2p_1/2_) and 162.0 eV (S 2p_3/2_), indicating that the sulphur is chemisorbed on the gold surface.^41^ The second doublet peak can be observed at 163.8 eV and 165.0 eV, which is assignable to the S–C bond in the biotin moiety.^42,43^ The N 1s spectrum (Fig. 3b) can be deconvoluted into two peaks, which support the presence of the peptide on the surface. The first peak centred at 400.5 eV is attributed to amino (NH_2_) and amide (CONH) moieties. The second peak centred at 402.3 eV is ascribed to protonated amino groups.^44^ Note that no nitrogen peak was observed for pure **C11TEG** SAMs (Fig. S1†). The C 1s spectrum (Fig. 3c) can be deconvoluted into three peaks, which are attributed to five different binding environments. The peak at 285.1 eV is attributed to C–C bonds,^45^ while the peak at 286.4 eV corresponds to C 1s of the three binding environments of C–S, C–N and C–O.^45^ The third and smaller peak (288.7 eV) is assigned to the C 1s photoelectron of the carbonyl moiety, C=O.^45^ The O 1s spectrum (Fig. 3d) is de-convoluted into two different peaks, corresponding to two different binding environments, arising from the C–O (533.2 eV) and C=O (532.0 eV) bonds.^45^ Furthermore, from integrating the area of the S 2p and N 1s peaks and taking into consideration that the **biotin-4KC** oligopeptide consists of 11 N atoms and 2 S atoms and **C11TEG** has no N and 1 S atom only, it was possible to infer that the ratio of **biotin-4KC** : **C11TEG** on the surface is 1 : 8 ± 4.

**Fig. 4:**
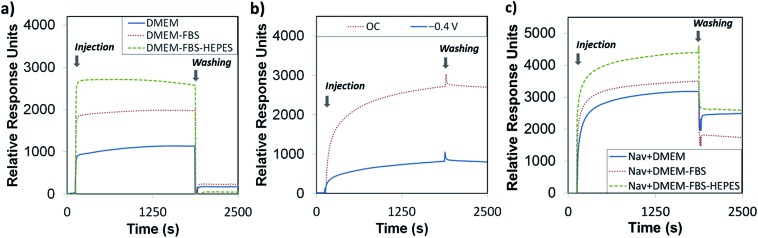
SPR sensorgram traces for (a) the interaction of **biotin-4KC**:**C11TEG** SAMs with different complex media – DMEM, DMEM–FBS, and DMEM–FBS–HEPES; (b and c) the binding of neutravidin (Nav) to the **biotin-4KC**:**C11TEG** mixed SAMs. (b) The mixed SAMs were exposed to neutravidin under OC conditions and an applied negative potential (–0.4 V). (c) The mixed SAMs were exposed to neutravidin in either DMEM, DMEM–FBS or DMEM–FBS–HEPES. After neutravidin binding for 30 min, the surfaces were washed with PBS for 10 min to remove any non-specifically adsorbed material. The two arrows in the graphs indicate the point of injection of neutravidin either in PBS or media and PBS washing buffer, respectively.

At this stage, it was important to assess if the **biotin-4KC**:**C11TEG** was inert to non-specific binding events while capable of maximum specific binding to neutravidin. We began our inquiry by looking at the resistance of the mixed SAMs to non-specific adsorption by exposing them to the three media, DMEM, DMEM–FBS, and DMEM–FBS–HEPES and analysed the adsorption response by SPR. In the SPR experiments, the SAMs were exposed to an initial flow of PBS solution, to establish the baseline, followed by an injection (first arrow on left in Fig. 4a) of either DMEM, DMEM–FBS or DMEM–FBS–HEPES media into the SPR flow cell for 1800 s (*i.e.* 30 min) at a flow rate of 8 μl min^–1^. The SPR flow cell was then flushed with PBS to remove any physisorbed material, at *t* = 1800 s (second arrow in Fig. 4a). Upon initial injection of the three different media over the **biotin-4KC**:**C11TEG**, there was a rapid response due to differences in the refractive indices of the PBS buffer and three media solutions, followed by smaller changes over the 30 min exposure to media. On washing with PBS buffer (*t* = 1800 s, arrow on the right in Fig. 4a) into the flow cell, there was an immediate drop in the SPR signal due to the change in the bulk refractive index. The **biotin-4KC**:**C11TEG** monolayer exhibits high resistance to non-specific adsorption from all three media, with SPR responses of less than 230 response units (RU). These levels of non-specific binding on **biotin-4KC**:**C11TEG** SAMs are equivalent to less than 23 ng cm^–2^ of adsorbed proteins, which compares well with those seen on other anti-fouling SAM systems.^39^

To demonstrate the ‘uninhibited’ binding capacity (*i.e.* the binding capacity of the surface without proteins, amino acids, glucose and vitamins in the subphase) of **biotin-4KC**:**C11TEG** SAM to neutravidin, SPR experiments were performed by injecting the neutravidin in PBS (arrow on left in Fig. 4b) to the mixed SAM, and monitoring the SPR response for 1800 s (OC trace), before washing with PBS (arrow on right in Fig. 4b), noting only a small drop in the SPR signal upon washing. The binding capacity is defined as the difference in the SPR response units between the beginning of injection of protein and the end of washing with PBS. As highlighted in Fig. 4b (OC trace), specific binding of neutravidin to the **biotin-4KC**:**C11TEG** SAM resulted in a binding capacity of ∼2700 RU. These results indicate that the longer **C11TEG** shielding component does not interfere with the binding capacity of the biotin ligand.

In addition, the switching efficiency was assessed in terms of the **biotin-4KC**:**C11TEG** mixed SAM ability to control the binding between the surface-appended biotin and the neutravidin from the ‘uninhibited’ PBS solution after the SAM surface had been preconditioned with a –0.4 V potential. Previously, we have demonstrated that the bio-inactive “OFF” state can be effected by application of –0.4 V, while not affecting the SAM integrity.^25^ The neutravidin in PBS was injected (arrow on left in Fig. 4b –0.4 V trace) with the –0.4 V potential being applied for 30 min, after which the surface was rinsed with PBS (again noting the small drop in SPR response after PBS washing). The switching efficiency (*S*_Ef_) was defined as the percent difference between the binding capacity at open circuit conditions (BC_OC_) and the binding capacity at –0.4 V (BC_–0.4 V_) divided by BC_OC_:
1

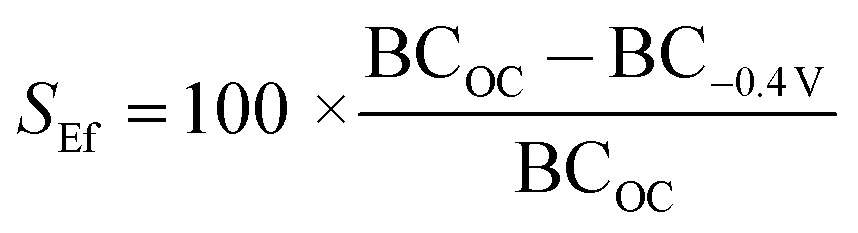




Thus, given the binding capacity of 2700 RU was observed under OC conditions, whereas a negative potential of –0.4 V induced a large reduction in binding affinity, with the SPR response decreasing to 800 RU, the switching efficiency of the **biotin-4KC**:**C11TEG** mixed SAMs in PBS was ∼70%, thereby demonstrating the suitability of the **biotin-4KC**:**C11TEG** mixed SAM for further experiments.

In order to evaluate the binding capacity of the **biotin-4KC**:**C11TEG** mixed SAM under complex biological conditions, neutravidin binding to the biotin ligand on the mixed SAM was monitored in the presence of the three media (Fig. 4c, open circuit conditions). In the SPR experiments, the mixed SAMs were exposed to a flow of PBS, to establish the baseline, followed by an injection of neutravidin in either DMEM, DMEM–FBS or DMEM–FBS–HEPES into the SPR flow cell for 30 min. The SPR flow cell was then flushed with PBS to leave only the specifically bound neutravidin on the biotinylated mixed SAM. Following rinsing with PBS, the final SPR signal associated with neutravidin and DMEM (2500 RU) and neutravidin and DMEM–FBS–HEPES (2600 RU) was comparable to the response associated with neutravidin in PBS (2700 RU), whereas a decrease in neutravidin binding was observed when the mixed SAM was exposed to neutravidin in DMEM–FBS (1800 RU) (Fig. 4c).

The differences observed in the representative SPR sensorgrams suggest that the presence of FBS to some extent interfered with the binding of neutravidin. The serum proteins are most likely non-specifically adsorbing to the surface alongside the specific adsorption of the neutravidin, and hence block some of the biotin moieties, not allowing them to bind to the neutravidin. Interestingly, the presence of HEPES surfactant in the DMEM–FBS–HEPES solution allowed more neutravidin to bind to the biotinylated surface, which correlates well with the earlier reports which state that protein adsorption depends upon, among other factors, the medium in which the protein is found.^46^ In this case, the HEPES may be coating the serum proteins in the FBS, which in turn is inhibiting them from binding to the surface, and hence not blocking the biotin moieties from binding to the neutravidin.

**Fig. 5:**
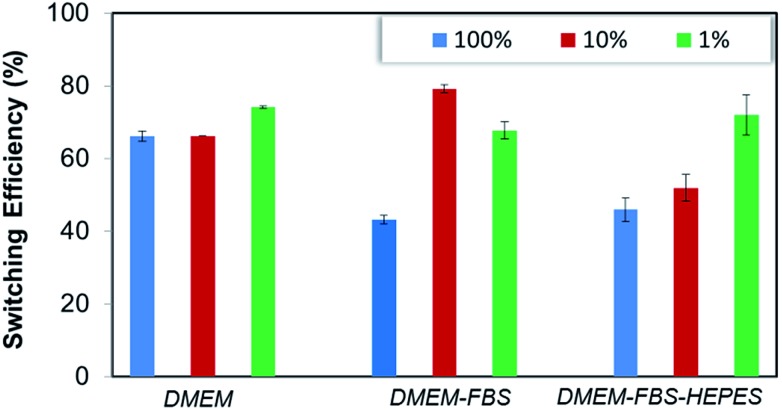
Switching efficiency, as determined by SPR analysis under OC conditions and an applied negative potential (–0.4 V), on **biotin-4KC**:**C11TEG** mixed SAMs which were exposed to neutravidin in (a) DMEM, DMEM–FBS, DMEM–FBS–HEPES. The switching efficiency of the different media was tested at different dilutions in PBS buffer. Error bars show standard deviations among three different substrates.

The **biotin-4KC**:**C11TEG** mixed SAMs were further studied with respect to the switching efficiency in the presence of the three different media (undiluted medium is termed 100% in Fig. 5). Similar efficiency to PBS was reached when DMEM was employed as the control media (67%), whereas the presence of DMEM–FBS and DMEM–FBS–HEPES during the switching process had induced a drop off in efficiency to values close to 45%.

In order to adequately represent the factors influencing the switching ability, the oligopeptide mixed SAMs were tested using the three media (*i.e.* DMEM, DMEM–FBS and DMEM–FBS–HEPES) at different dilutions in PBS buffer (Fig. 5). Interestingly, a dilution in PBS to 10% of the different media had no effect on the switching efficiency of DMEM, while it had improved the efficiency of the DMEM–FBS and DMEM–FBS–HEPES systems at different rates. As seen in Fig. 5, switching efficiency increased roughly 35% as the DMEM–FBS concentration decreases from 100% (undiluted medium) to 10%, whereas the same decrease in concentration for the DMEM–FBS–HEPES has led to no differences in efficiency within the error. The analysis of the effect of concentration on the switching efficiency of the **biotin-4KC**:**C11TEG** mixed SAMs also indicate that 1% media solutions have negligible effect on the switching efficiency, with all three media showing values similar to those observed for PBS, *i.e.* of approximately 70%. At this point, it should be stressed that even though the switching is partly compromised when compared to very diluted media (*i.e.* 1%) or pure buffer, the level of switching in 10% and 100% media is still relatively high and in all instances is higher than 45%.

**Fig. 6:**
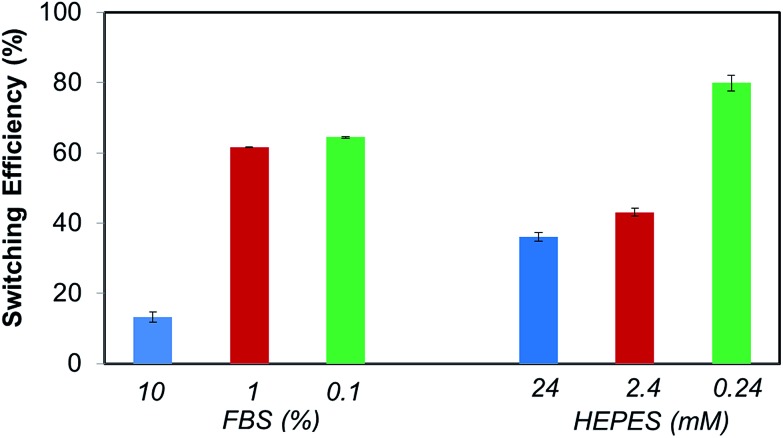
Switching efficiency, as determined by SPR analysis under OC conditions and an applied negative potential (–0.4 V), on **biotin-4KC**:**C11TEG** mixed SAMs which were exposed to neutravidin in FBS and HEPES. The switching efficiency of the different media was tested at different dilutions in PBS buffer. Error bars show standard deviations among three different substrates.

Also relevant, these dilutions studies indicate that FBS and HEPES might have distinct effects in the switching ability of the electrically switchable SAM surface. Thus, in order to further delineate the respective roles of FBS and HEPES in the switching process, SPR switching studies were performed with these two individual components. FBS and HEPES solutions in PBS were used at the same concentration as in the DMEM–FBS and DMEM–FBS–HEPES (*i.e.* 10% FBS and 24 mM HEPES). As before, the baseline for the **biotin-4KC**:**C11TEG** mixed SAM-modified gold chip was established using PBS, following which the neutravidin in either of the solutions mentioned above (*i.e.* 10% FBS and 24 mM HEPES) was introduced for 30 min. Subsequently, the surfaces were washed in PBS. These SPR experiments were conducted under OC conditions and –0.4 V in order to calculate the binding switching efficiency as described above, eqn (1). The binding switching efficiencies of the **biotin-4KC**:**C11TEG** mixed SAM in the presence of these 10% FBS or 24 mM HEPES are summarised in Fig. 6.

Interestingly, the switching behavior of the mixed SAM on 10% FBS has led to efficiencies around 15%, indicating that the switching effect was largely absent. Remarkably, this value can be significantly enhanced, rising to 45%, if the switching is performed using such FBS concentrations in the presence of HEPES as shown in Fig. 5. Nevertheless, it is clear that the presence of a significant amount of protein results in the partial inhibition of the switching process. This behaviour might be attributed to the interference of the FBS with the conformation changes in the oligopeptide. This reasoning is in line with the decreased specific binding capacity when the **biotin-4KC**:**C11TEG** mixed SAM was exposed to neutravidin in the presence of DMEM–FBS (Fig. 4c), showing that non-specific interactions between FBS and the oligopeptide mixed SAM caused interference with the specific binding between neutravidin and the surface-appended biotin.

From the HEPES experiments, some valuable information can also be gathered. The switching efficiency is also affected by the presence of HEPES, which has led to efficiencies values of 40%. HEPES contains both a very strong acid (sulfonic acid) and a relatively weak base (amine) and they are particularly prone to the formation of hydrogen bonds and electrostatic interactions with proteins as is seen in several protein crystal structures.^47,48^ From the aforementioned results, and on the basis of the previous literature of the interactions of the zwitterionic HEPES with proteins, we suggest that the ability of the HEPES molecule to form stable intermolecular interactions with the peptide might restrict the oligopeptide from electrostatically interacting with the negatively charged gold surface and change its conformation, resulting in a decrease in switching efficiency. The noted protein–HEPES interactions can also explain the reduction in non-specific FBS adsorption (Fig. 4c) when HEPES was present in the media. HEPES interactions with FBS might prevent non-specific interactions between FBS and **biotin-4KC**:**C11TEG** mixed SAM.

Dilution studies were also carried out using FBS and HEPES solutions. FBS and HEPES solutions in PBS were diluted to 10% and 1% of the original concentrations. The FBS solutions are designated as 1% FBS and 0.1% FBS, while the HEPES solutions are denominated as 2.4 mM and 0.24 mM HEPES (Fig. 6). Interestingly, the switching behavior of the mixed SAM on 1% and 0.1% FBS differed strikingly from that observed on 10% FBS. Switching efficiency was pronounced for 1% and 0.1% FBS, with values in the range of 60–65%, which are comparable to those observed for PBS. The HEPES dilutions experiments have also shown that the switching efficiency is strongly dependent on the concentration of HEPES, which by decreasing from 24 mM to 0.24 mM has led to a marked augmentation in switching efficiency from 35% to more than 75% (Fig. 6). No major differences were found between 24 mM and 2.4 mM HEPES, which remained in the range of 35–45%. Taken together, these results clearly show the importance of selecting a buffer that has a minimal impact on the switching ability of the oligopeptide or any other switchable surface system that bases its switching mechanism on a charged molecular backbone or end group. Careful control of the media composition ensures that the switching based on the oligopeptide can achieve levels of efficiency as high as 70%.

## Conclusions

**Fig. 7:**
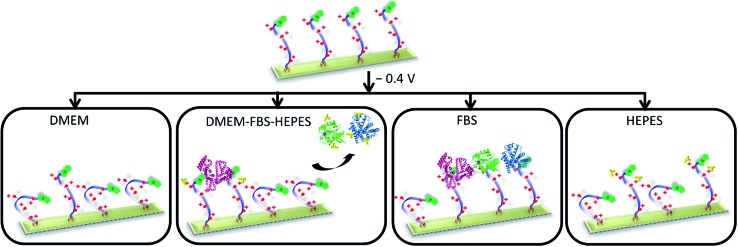
Schematic showing that the characteristics of the media influence the performance of the switching of the electro-switchable oligopeptides. DMEM exhibits similar switching behaviour as PBS, whereas DMEM–FBS–HEPES induces a drop in switching efficiency. However, the switching ability is higher than when only FBS is used. We propose that the presence of HEPES in the DMEM–FBS–HEPES media allows the formation of hydrogen bonds and electrostatic interactions between HEPES and the serum proteins, leading to a decrease in the interactions between the serum proteins and the switchable surface. High concentrations of HEPES also inhibit to a certain extent the switching of the oligopeptides likely as a result of intermolecular interactions between HEPES and the oligopeptides. Not to scale (see Fig. 1 for the description of the cartoons). The oligo(ethylene glycol) thiols have been removed for clarity.

While substantial attention has been directed to construction and performance of biological switchable surfaces in simple biological systems, less effort has been directed to developing and understanding surfaces capable of switching under more realistic biological environments. To the best of our knowledge, this is the first study to investigate and address such scientific issues and challenges associated with the underpinnings of biological switchable surfaces. In this work, we merged an approach for producing well-defined SAMs that prevent non-specific binding with the ability to electrically switch the SAM to allow control over biomolecular interactions under complex biological matrixes. Particularly, this SAM system can be dynamically modulated by an electrical potential under different commonly used biological media, ranging from DMEM to DMEM supplemented with FBS and HEPES. The work demonstrated that the performance of the switching on the electro-switchable oligopeptide is sensitive to the characteristics of the media, and in particular, its protein concentration and buffer composition (Fig. 7). The design of an electrical stimuli-responsive surfaces and their operation under complex biological conditions must properly take these issues into account to ensure maximum switching performance. This study will no doubt be useful in developing more realistic dynamic extracellular matrix models and is certainly applicable in a wide variety of biological and medical applications.
