# Supplementary material for: Switching specific biomolecular interactions on surfaces under complex biological conditions
Source: Analyst. 2014 Sep 2;139(21):5400–8. doi: 10.1039/c4an01225a (PMC4184031; doi:10.1039/c4an01225a)
Supplement: Supplementary file 1 [file AN-139-C4AN01225A-s001.pdf]

Electronic Supplementary Material (ESI) for Analyst.
This journal is © The Royal Society of Chemistry 2014

Supplementary Data

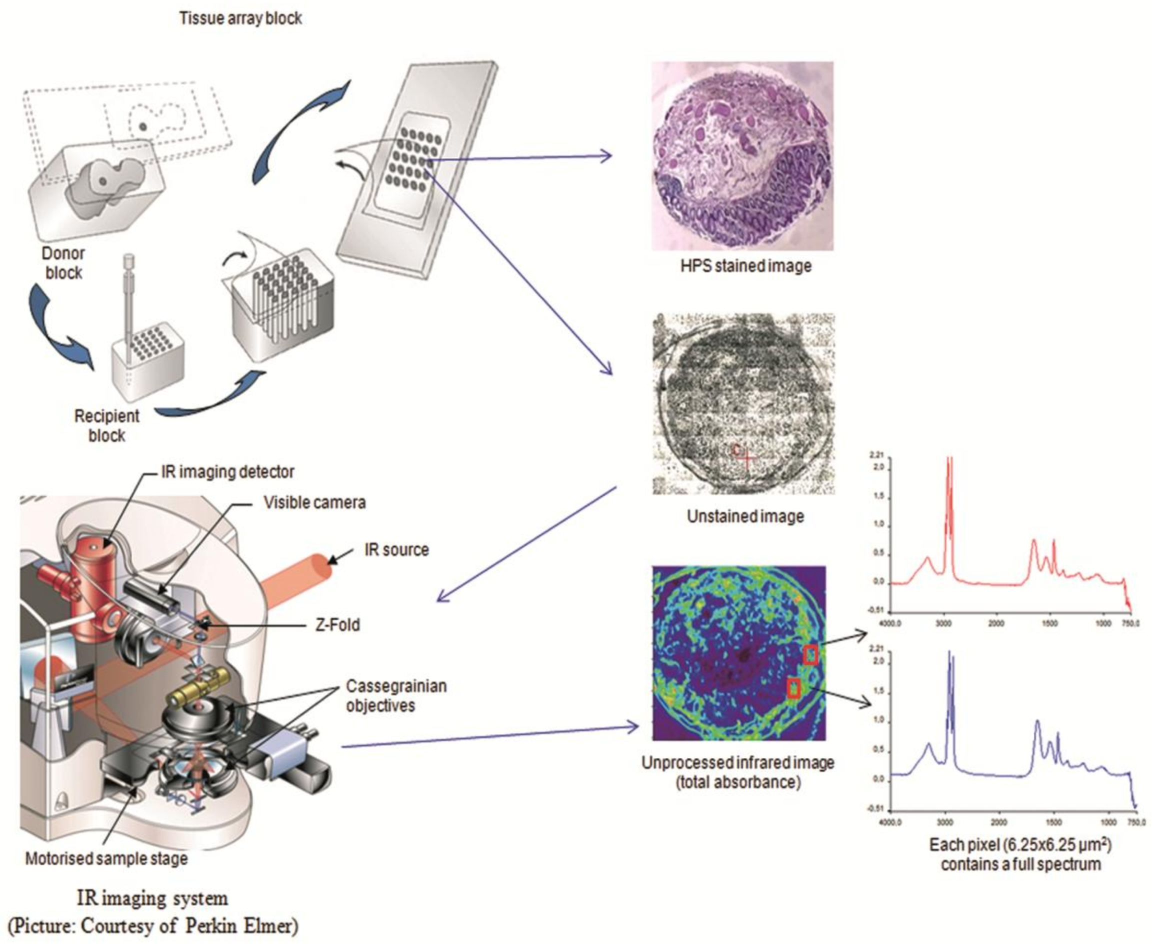

Supplementary Figure 1

Schematic representation of infrared spectral imaging applied to paraffinized tissue arrays.

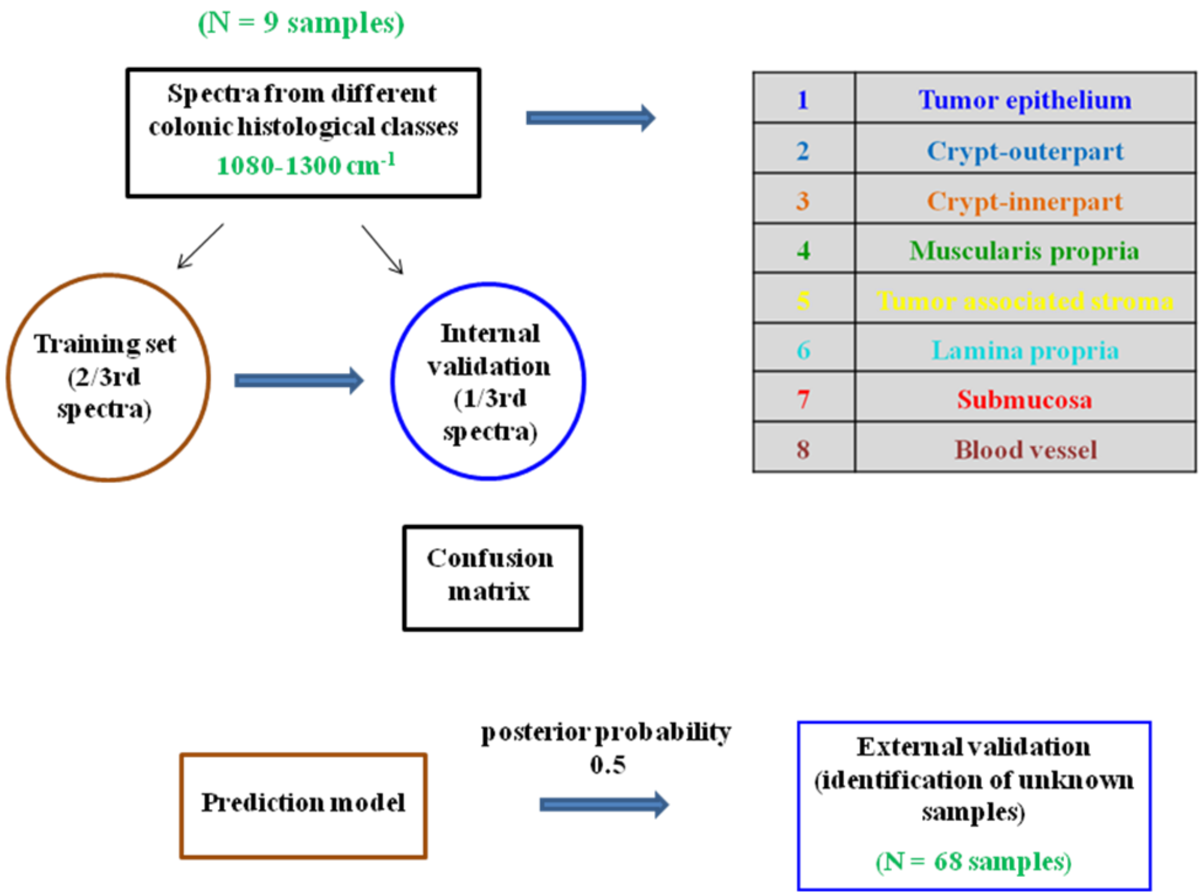

Supplementary Figure 2

Schematic representation of construction and application of the prediction model based on linear discriminant analysis.

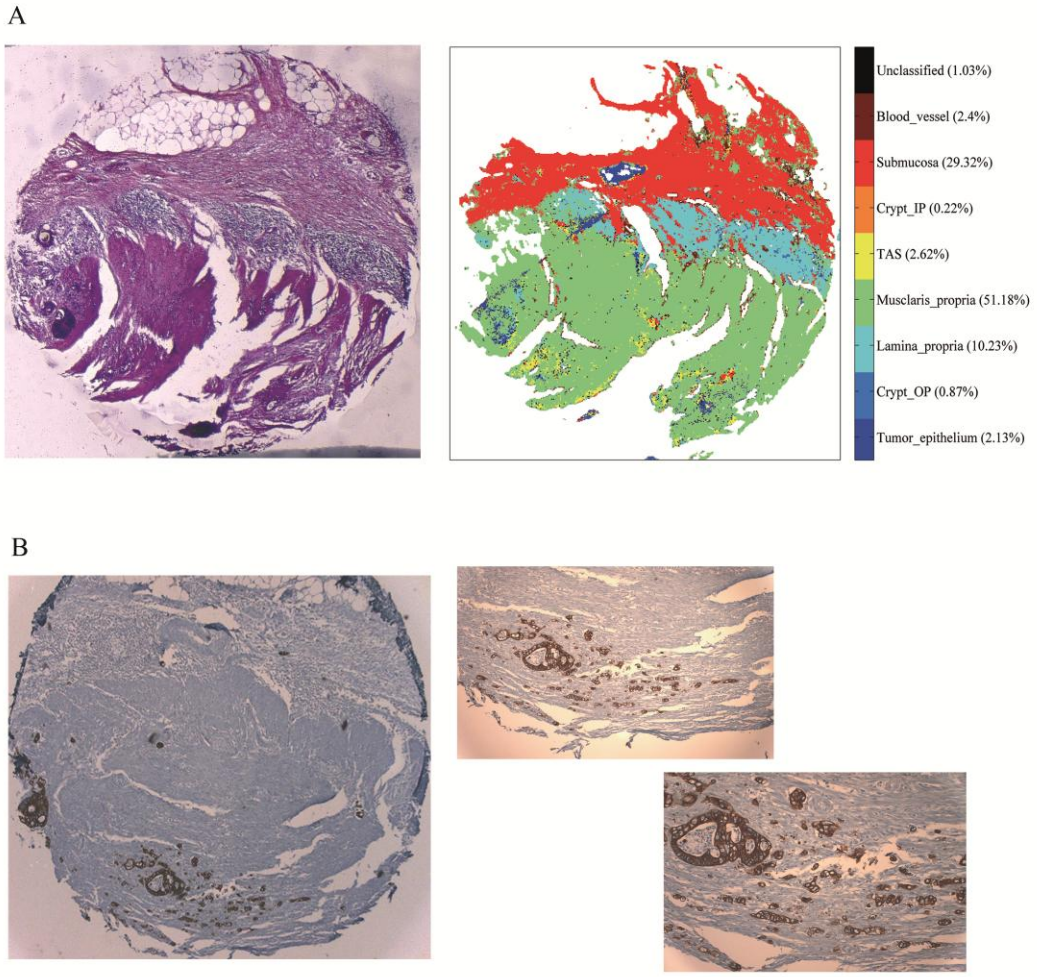

Supplementary Figure 3

Identification of tumor budding in an unknown colonic tissue. Left panel (A): HPS stained colon tissue (Supplementary Table S1, sample # 9A); Right panel (A): Infrared spectral predicted images. Left panel (B): KL 1 immuno-stained image; Right panel (B): zoomed in area of the same image with positive staining. The sample is a moderately differentiated colon adenocarcinoma with tumor-budding branching out into the stroma. The presence of even very few tumor cells sparsely visible in the HPS image are identified and predicted by the model, as can be verified from the immuno-stained image. The HPS and the IHC images are at 5X magnification, and the zoomed images are at 10 X (top) and 20 X (bottom) magnifications respectively.

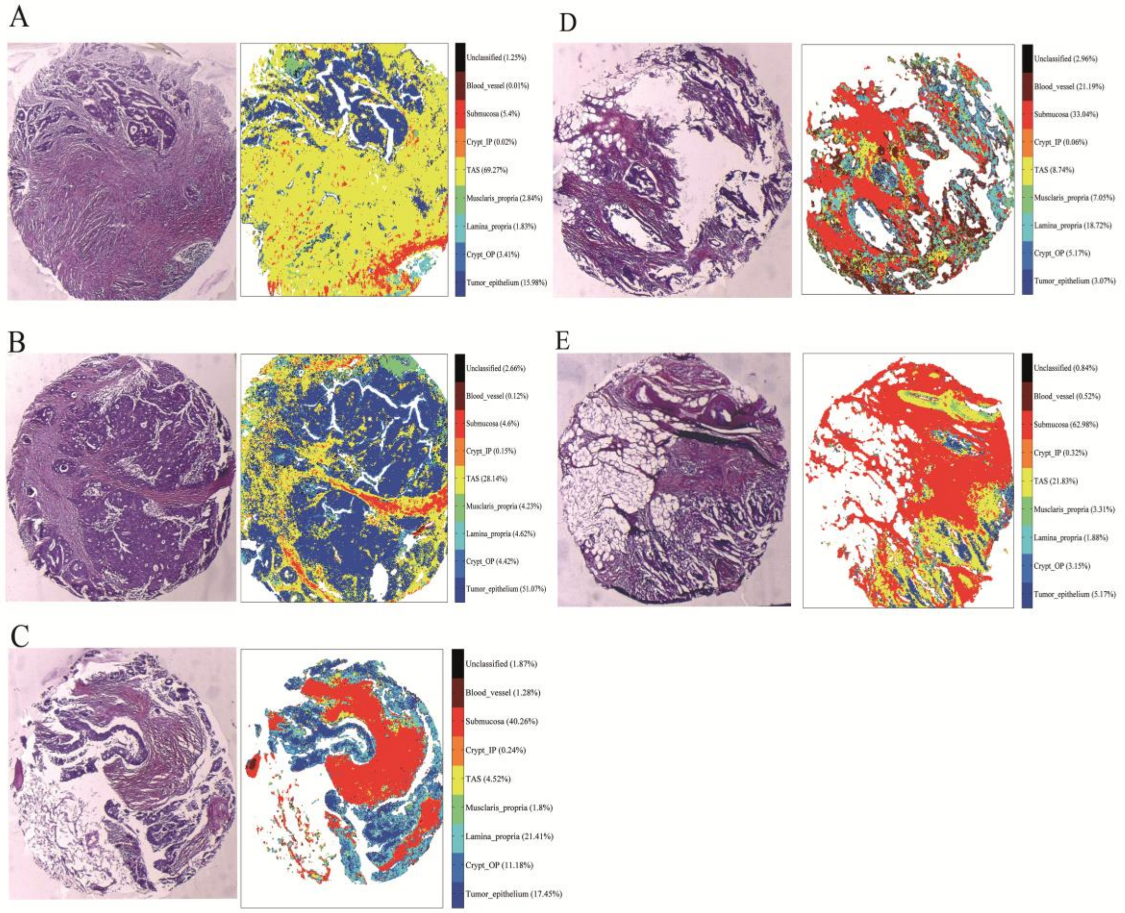

Supplementary Figure 4

Tumor stroma geographical proximity. Left panel: HPS stained colon tissues; Right panel: Infrared spectral predicted images. The samples are moderately differentiated colonic adenocarcinoma with its associated stroma with infiltration into the adjacent connective tissue (Supplementary Table S1, sample # 11A, 11C, 12A, 13A, and 15A). Along with tumor identification, the nature of the connective tissue into which the tumor has infiltrated is also identified. The HPS images are at 5X magnification.

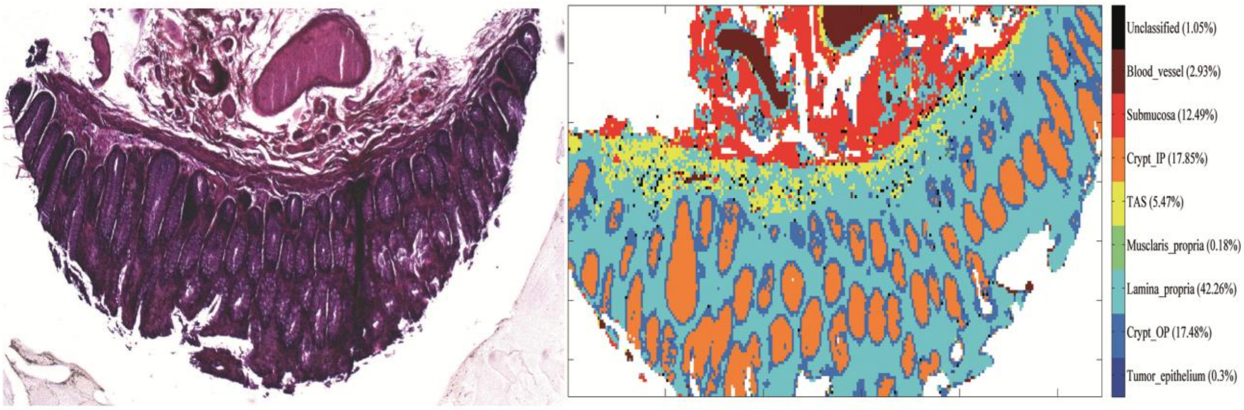

Supplementary Figure 5

Confusion between muscularis mucosa and stroma. Left panel: HPS stained colon tissues; Right panel: Infrared spectral predicted image. The sample is a non-tumoral colonic tissue (Supplementary Table S1, sample # 27) in which all the normal characteristics of the normal colonic architecture are well-identified, however the thin layer of muscularis mucosa is identified as tumor associated stroma by the prediction model seen as yellow pixels. The HPS image is at 5X magnification.

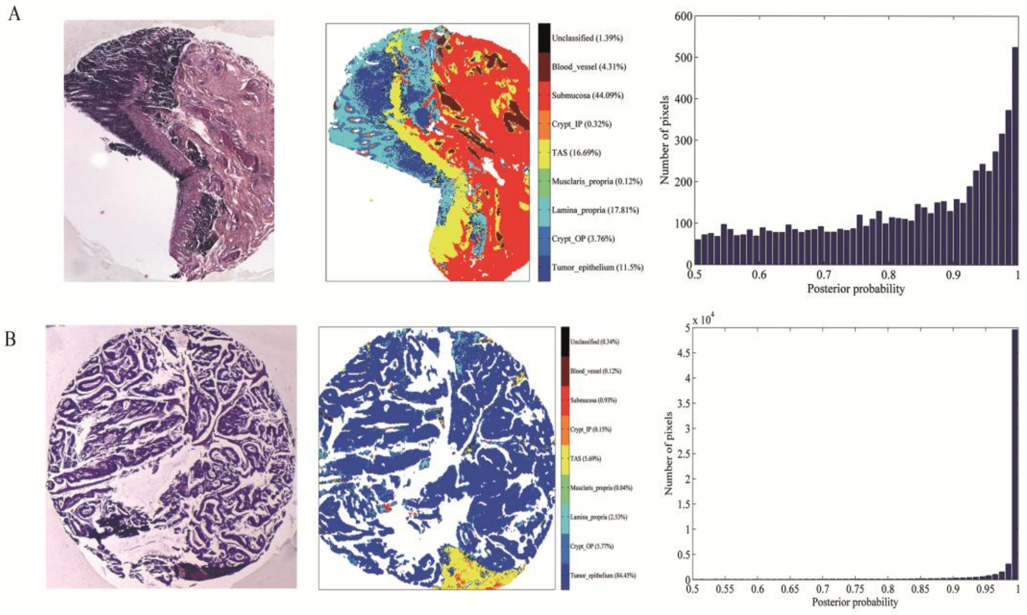

Supplementary Figure 6

Histogram for tumor pixel attribution in non-tumoral and tumoral sample (Supplementary Table S1, sample # 32 and 8C). Left panel: HPS stained colon tissues; Middle panel: Infrared spectral predicted image; Right panel: Histograms for the tumor pixel attribution. A is a non-tumoral colonic tissue with inflammatory signatures. These pixels (depicted as tumor pixels in A) have lesser posterior probability values compared to B which is a moderately differentiated colon adenocarcinoma in which the tumor pixels have the highest posterior probability values.

Supplementary Table 1

Sample details

Patient No.	Sex/Age	Tumor			Normal	Colon Carcinoma location	TNM classification	Non-tumoral tissue location
		Front (A)	Lateral (B)	Middle (C)	Distant (D)			
1	F, 67				TG	L	T3N0	L
2	M, 71	**		TG	TG	L	T3 N0	L
3	F, 74	**	**		**	L	T3 N0	L
4	F, 48				LF	L	T3 N0	L
5	F, 61				**	R	T3 N0	R
6	M, 70	**			**	L	T3 N1	L
7	M, 76					L	T3 N1	L
8	F, 62				TG	L	T3 N0	L
9	F, 66					L	T3 N2	L
10	F, 72	**	TG		LF	R	T4 N0	R
11	M, 51				LF	R	T3 N1	R
12	F, 51		TG	TG	TG	L	T3 N1	L
13	M, 66					L	T3 N0	L
14	F, 57	**				L	T3 N1	L
15	F, 41		TG		LF	R	T3 N1	R
15	As above	LF				R	T3 N1	R
16	M, 73					L	T3 N2	L
17	F, 46				**	R	T4 N2	R
18	F, 91							R
19	F, 78							R
20	M, 41							R
21	M, 72							R
22	M, 54				LF			Peri-tumoral
23	M, 68				LF			Peri-tumoral
24	F, 82				LF			Peri-tumoral
25	F, 90				LF			Peri-tumoral
26	M, 64							Sigmoid
27	M, 64							Sigmoid
28	M, 82				LF			Sigmoid
29	M, 69				LF			Sigmoid
30	F, 53							Sigmoid
31	F, 79							R
32	F, 73				LF			Sigmoid

(shaded)	Analyzed
**	Not analyzed
TG	Training group
LF	Inflammation
L	Left
R	Right

Supplementary Table 2

Infrared spectral peak attribution:

Attribution of infrared spectral peaks (the most discriminant wavenumbers identified using the Mann-Whitney *U* test as shown in figure 5) for the compared classes in the selected infrared spectral region from 1080 cm^{-1} to 1300 cm^{-1}.

Table 2: Infrared spectral peak attribution (cm^{-1}) (2, 3, 11, 30, 48, 49)					
Normal epithelium - tumor epithelium		Tumor epithelium - Tumor associated stroma		Connective tissue - Tumor associated stroma	
Peak position	Biomolecular attribution	Peak position	Biomolecular attribution	Peak position	Biomolecular attribution
1082	PO_2^- symmetric stretch of nucleic acids				
		1214	Collagen	1242	Collagen
1240	PO_2^- asymmetric stretch of nucleic acids	1280		1280	
1155	C-O stretch of Carbohydrates				
1160	H-bonded C-O stretch of Proteins				
1176	non-H-bonded C-O stretch of Proteins				

2. C. Conti, P. Ferraris, E. Giorgini, C. Rubini, S. Sabbatini, G. Tosi, J. Anastassopoulou, P. Arapantoni, E. Boukaki, S. Konstadoudakis, T. Theophanides and C. Valavanis, *J. Mol. Struct.*, 2008, **881**, 46–51.

3. H. Miyoshi, M. Oka, K. Sugi, O. Saitoh, K. Katsu and K. Uchida, *Intern. Med.*, 2000, **39**, 701–706.

11. M. J. German, A. Hammiche, N. Ragavan, M. Tobin, L. J. Cooper, S. S. Matanhelia, A. C. Hindley, C. M. Nicholson, N. J. Fullwood, H. M. Pollock and F. L. Martin, *Biophys. J.*, 2006, **90**, 3783–3795.

30. M. Khanmohammadi, M. A. Ansari, A. B. Garmarudi, G. Hassanzadeh and G. Garoosi, *Cancer Invest.*, 2007, **25**, 397–404.

48. S. L. Patrick, T. T. Wong and H. M. Yazdi, *Appl. Spectrosc.*, 1993, **47**, 1830–1836.

49. L. Chen, H. Y. N. Holman, H. Zhao, H. A. Bechtel, M. C. Martin, C. Wu and S. Chu, *Anal. Chem.*, 2012, **84**, 4118–4125.
